# Neurogranin and Neurofilament Light Chain as Preclinical Biomarkers in Scrapie

**DOI:** 10.3390/ijms23137182

**Published:** 2022-06-28

**Authors:** Marina Betancor, Sonia Pérez-Lázaro, Alicia Otero, Belén Marín, Inmaculada Martín-Burriel, Kaj Blennow, Juan José Badiola, Henrik Zetterberg, Rosa Bolea

**Affiliations:** 1 Centro de Encefalopatías y Enfermedades Transmisibles Emergentes, Universidad de Zaragoza, IA2, IIS Aragon, 50009 Zaragoza, Spain; mbetancorcaro@gmail.com (M.B.); soniaperez@unizar.es (S.P.-L.); belenm@unizar.es (B.M.); minma@unizar.es (I.M.-B.); badiola@unizar.es (J.J.B.); rbolea@unizar.es (R.B.); 2 Laboratory of Biochemical Genetics (LAGENBIO), Faculty of Veterinary, University of Zaragoza, Miguel Servet 177, 50013 Zaragoza, Spain; 3 Centro de Investigación Biomédica en Red de Enfermedades Neurodegenerativas (CIBERNED), Instituto Carlos III, 28220 Madrid, Spain; 4 Department of Psychiatry and Neurochemistry, Institute of Neuroscience & Physiology, The Sahlgrenska Academy, University of Gothenburg, 405 30 Mölndal, Sweden; kaj.blennow@neuro.gu.se (K.B.); henrik.zetterberg@clinchem.gu.se (H.Z.); 5 Clinical Neurochemistry Laboratory, Sahlgrenska University Hospital, 413 45 Mölndal, Sweden; 6 Department of Neurodegenerative Disease, University College LondonInstitute of Neurology, Queen Square, London WC1N 3BG, UK; 7 UK Dementia Research Institute, University College London, London WC1E 6BT, UK; 8 Hong Kong Center for Neurodegenerative Diseases, Clear Water Bay, Hong Kong, China

**Keywords:** prion, prion diseases, scrapie, biomarkers, neurodegeneration, neurogranin, neurofilament light chain

## Abstract

Prion diseases are diagnosed in the symptomatic stage, when the neuronal damage is spread throughout the central nervous system (CNS). The assessment of biological features that allow the detection of asymptomatic cases is needed, and, in this context, scrapie, where pre-symptomatic infected animals can be detected through rectal biopsy, becomes a good study model. Neurogranin (Ng) and neurofilament light chain (NfL) are proteins that reflect synaptic and axonal damage and have been studied as cerebrospinal fluid (CSF) biomarkers in different neurodegenerative disorders. In this study, we evaluated Ng and NfL both at the protein and transcript levels in the CNS of preclinical and clinical scrapie-affected sheep compared with healthy controls and assessed their levels in ovine CSF. The correlation between these proteins and the main neuropathological events in prion diseases, PrP^Sc^ deposition and spongiosis, was also assessed. The results show a decrease in Ng and NfL at the protein and gene expression levels as the disease progresses, and significant changes between the control and preclinical animals. On the contrary, the CSF levels of NfL increased throughout the progression of the disease. Negative correlations between neuropathological markers of prion disease and the concentration of the studied proteins were also found. Although further research is needed, these results suggest that Ng and NfL could act as biomarkers for neurodegeneration onset and intensity in preclinical cases of scrapie.

## 1. Introduction

Transmissible Spongiform Encephalopathies (TSEs), also known as prion diseases, are fatal neurodegenerative disorders that include the different forms of Creutzfeldt–Jakob disease (CJD) in humans, bovine spongiform encephalopathy (BSE) in cattle, and scrapie in sheep and goats, among others [1]. They are caused by prions, which induce the conversion of the cellular prion protein (PrP^C^) into a misfolded form known as PrP^Sc^, which accumulates in the central nervous system (CNS), leading to spongiform degeneration and neuronal loss [2]. Hitherto, in humans, prion diseases can only be identified and diagnosed in symptomatic patients [3]; therefore, it is not known whether cerebrospinal fluid (CSF) tests would detect non-symptomatic patients of CJD. In sporadic CJD (sCJD), characterized by rapidly progressive dementia and ataxia [4], two established markers in the routine diagnostics are 14-3-3 and total tau in CSF, both considered as markers of neuronal damage [5]. Moreover, the real-time quaking-induced conversion assay (RT-QuIC), which directly detects the pathogen, is used as a highly specific and sensitive test in CSF [6]. In sheep scrapie, contrary to human cases, non-asymptomatic prion-infected animals can be easily detected by rectal biopsy [7]. A previous study [8] demonstrated that CSF 14-3-3, tau, and PrP seeding activity (RT-QuIC) tests can also be useful in the identification of cases of scrapie, even in preclinical stages. Moreover, this study found the 14-3-3 and tau concentrations to be higher in the CSF of preclinical animals compared with controls, suggesting that the underlying neurodegenerative mechanisms in prion diseases could be occurring in asymptomatic stages.

Neurogranin (Ng, formerly named p17, also known as RC3 and BICKS) is a 78 amino acid post-synaptic protein with a molecular weight of ~12 kDa [9,10]. This protein is known to be principally expressed in the telencephalon of adult mammals, especially in the cerebral cortex, hippocampus, and striatum [11], as a neuronal postsynaptic protein, which is specifically located in the neuronal soma and dendrites [12]. Ng intracellularly binds to calmodulin (CaM) [13] and phosphatidic acid (PA) [14], playing an important role in long-term potentiation and enhancing synaptic plasticity and functioning [9], which are growing concerns in the pathogenesis of several neurological diseases [15,16,17,18]. In mice, aging has been associated with a reduction in Ng expression. which has been implicated in age-related cognitive impairment [19], and Ng knockout mice have presented deficits in spatial memory and long-term potentiation [20], whereas the upregulation of Ng can restore and strengthen neuron connections and improve cognitive deficit [21,22]. Blennow et al., (2019) [18], studied Ng as a neuronal damage marker in symptomatic CJD and Alzheimer’s disease patients (AD), and found that the CSF Ng levels in CJD were increased compared with healthy controls and even with AD, thereby representing a good biomarker to differentiate CJD from AD. Ng expression in brain tissue was decreased in clinical CJD cases compared with controls, whereas its levels in CSF were higher in clinical cases. This results in a negative correlation between the protein concentrations in brain tissue and CSF, i.e., a potential mobilization of the protein from degenerating dendrites into the CSF. Moreover, in CJD, the CSF Ng concentration did not differ between the early and late stages of the disease, supporting that synaptic damage is an early event in prion diseases.

Neurofilaments (Nfs) are proteins of the cytoskeleton principally located in large, myelinated neurons. They play an important role in neurons, conferring structural stability to the axons, and are composed of three different subunits: neurofilament light chain (NfL, 68 kDa), neurofilament medium chain (NfM, 160 kDa), and neurofilament heavy chain (NfH, 200 kDa) [23]. NfL is the most abundant and soluble subunit of Nfs, which makes it the most reliable Nfs subunit to be measured in biological fluids. When neuronal damage occurs, NfL is released to the interstitial fluid, which communicates with the CSF and the blood. Therefore, studying NfL as a biomarker for neurological diseases has gained attention, and this protein has been reported to be increased in blood and CSF in a variety of neurological disorders, such as AD, amyotrophic lateral sclerosis (ALS), frontotemporal dementia (FTD), or multiple sclerosis (MS) [24]. Regarding prion diseases, NfL has been found to be increased in the CSF and blood of CJD clinical cases [25,26,27]. Moreover, NfL appears to be increased in the serum of preclinical sheep, pinpointing it as a potential biomarker for scrapie [28]. However, further studies on NfL’s behavior through the pathogenesis of prion diseases and, particularly, on its concentrations in preclinical cases are needed.

As previously mentioned, currently, in humans, prion diseases are diagnosed in the symptomatic stage [3]. At this stage, the neuronal damage is already massive, and the benefit of possible treatments would be limited. Hence, the assessment of biological features allowing the detection of asymptomatic cases is needed. In this context, scrapie, where preclinical animals can be detected through rectal biopsy [4], becomes a good model to evaluate the biomarkers of naturally occurring prion disease in its early stages. Since synaptic and axonal damage are believed to be early pathogenic events in prion diseases [29,30], biomarkers that reflect early dysfunction at these levels might be valuable for early diagnosis. The CSF and the CNS are directly in contact, the former reflecting the latter’s biochemistry and pathophysiology status; therefore, potentially promising biomarkers might be studied in both nervous tissue and the CSF. Thus, our hypothesis is that the markers of synaptic and axonal damage will be reduced in brain tissue and increased in the CSF as the disease progresses, and that this change will be noticeable, even in the preclinical stages, reflecting the neuropathological events occurring in prion diseases. Therefore, the objective of this study is to evaluate Ng and NfL in the brain tissue and CSF of preclinical and clinical sheep naturally affected with scrapie, and compare the results with those obtained in healthy controls.

## 2. Results

### 2.1. Ng Expression Is Downregulated in Scrapie-Infected Sheep

**Figure 1:**
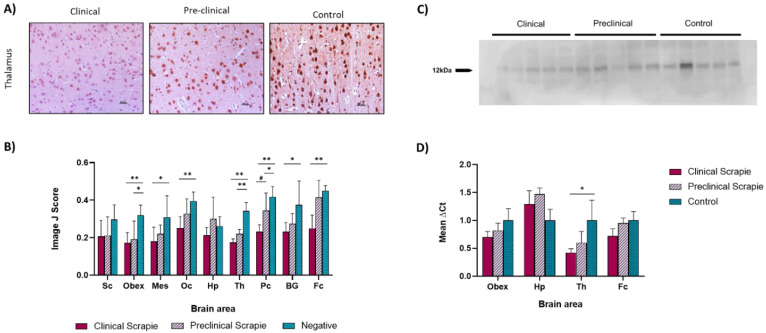
Assessment of Ng expression in clinical and preclinical scrapie-infected sheep and control sheep. (**A**) Representative images of Ng immunostaining in the thalamus from clinical (*n* = 8), preclinical (*n* = 5), and control (*n* = 8) sheep, where cytoplasmic labeling is observed. (**B**) Evaluation of Ng staining in the brains of clinical, preclinical, and control animals using Image J software in the spinal cord (Sc), obex, mesencephalon (Mes), occipital cortex (Oc), hippocampus (Hp), thalamus (Th), parietal cortex (Pc), basal ganglia (BG), and frontal cortex (Fc). Control sheep showed the highest scores in most of the assessed areas (except the hippocampus), followed by preclinical animals, while clinical sheep displayed the lowest scores in all brain areas. Evaluation of differences between groups was performed using the one-way ANOVA test followed by the Bonferroni post hoc test (# *p* = 0.05, * *p* < 0.05, and ** *p* < 0.01). The data shown in the figure represent the mean and the standard error of the mean (mean ± SEM). A tendency toward significance was found in the parietal cortex between clinical and preclinical sheep. (**C**) Western blot analyses for Ng in the frontal cortex of different groups of sheep using the Ng36 antibody. Samples correspond to clinical, preclinical, and control sheep. The detection of a band at ~12 kDa confirms the specificity of the antibody. (**D**) mRNA expression profiles of the Ng-encoding gene in the obex, hippocampus, thalamus, and frontal cortex of clinical, preclinical, and control sheep. Relative expression levels are expressed as the mean ± standard deviation. Normalization of the results was performed using the expression of *SDHA* and *GAPDH* housekeeping genes. The expression values were determined using the 2^−∆∆Ct^ method, and differences between experimental groups were analyzed using the one-way ANOVA test, followed by the Bonferroni post hoc test (* *p* < 0.05).

An immunohistochemical assessment of Ng expression was performed in the frontal cortex, basal ganglia, parietal cortex, thalamus, occipital cortex, hippocampus, mesencephalon, obex, and spinal cord of the three groups of sheep (clinical, preclinical, and controls). The resulting immunostaining pattern was defined by a uniform intraneuronal labeling that affected most of the neurons and by a slight diffuse staining of the neuropil. This pattern was consistent in the three groups of sheep, though, in general, the control group displayed a higher number of stained cells along with higher immunostaining intensities, followed by the preclinical animals, and clinical sheep showed the weakest staining (Figure 1A).

The Ng immunolabeling scores were measured using Image J software and displayed a trend to be higher in the control group compared with the clinical and preclinical sheep for all of the studied brain areas, except for the hippocampus (Figure 1B). In addition, Ng immunostaining was more intense in preclinical sheep compared with the clinical animals in most brain areas. Statistically significant differences were detected between the negative and clinical animals in all areas except for the hippocampus and spinal cord. The negative and preclinical groups showed significant differences in the thalamus (*p* < 0.01), obex, and parietal cortex (*p* < 0.05). No differences were found between the preclinical and clinical animals, though a tendency was observed at the parietal cortex (*p* = 0.05). Figure S1 shows a schematic map of the brain displaying the degree of Ng upregulation. The specificity of the Ng36 antibody, which was used against Ng in sheep nervous tissue, was confirmed by Western blot in the frontal cortex of the preclinical, clinical, and control sheep. Ng36 revealed a single band at ~12 kDa, corresponding to Ng’s molecular weight (Figure 1C).

The expression of the Ng-encoding gene (*NRGN*) was analyzed by quantitative PCR in four brain areas (obex, hippocampus, thalamus, and frontal cortex) of the three sheep groups to determine their mRNA expression levels throughout the course of the disease. Figure 1D displays the mean ∆Ct values of *NRGN*. The preclinical and clinical animals displayed a tendency toward the downregulation of the gene compared with controls in three of the studied brain areas (obex, thalamus. and frontal cortex), this being stronger in the clinical group, thus reflecting a trend toward decreasing regulation as the disease progressed. Significant differences were detected between the clinical and control animals at the thalamus level (*p* < 0.05).

### 2.2. NfL Expression Is Downregulated in Most Brain Areas of Scrapie-Infected Sheep

**Figure 2:**
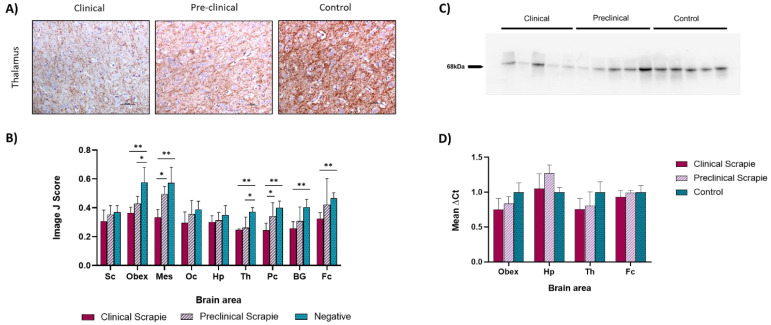
Study of NfL expression scrapie-infected and control sheep. (**A**) Images show the immunostaining pattern for NfL found in the thalamus of the different study groups: clinical (*n* = 8), preclinical (*n* = 5), and control (*n* = 8) sheep. Axonal and dendritic labeling can be observed. (**B**) Comparison of NfL Image J scores in the different studied brain areas of the three sheep groups: spinal cord (Sc), obex, mesencephalon (Mes), occipital cortex (Oc), hippocampus (Hp), thalamus (Th), parietal cortex (Pc), basal ganglia (BG), and frontal cortex (Fc). Control sheep exhibited the highest scores in all brain areas, followed by preclinical animals, while clinical sheep showed the lowest scores. Evaluation of differences between groups was performed using the one-way ANOVA test followed by the Bonferroni post hoc test (* *p* < 0.05 and ** *p* < 0.01). Data represent the mean ± SEM. (**C**) Western blot analyses for NfL in the frontal cortex of sheep. Samples corresponding to the frontal cortexes of clinical, preclinical, and negative sheep were analyzed. A single band at ~68 kDa was detected, confirming the specificity of the anti-NfL antibody. (**D**) Figure showing the mRNA expression profiles of the NfL-encoding gene in the obex, hippocampus, thalamus, and frontal cortex of the three groups of sheep. Relative expression levels are expressed as the mean ± standard deviation. The results were normalized using the expression of *SDHA* and *GAPDH* housekeeping genes. The expression values were determined using the 2^−∆∆Ct^ method, and differences between experimental groups were assessed using the one-way ANOVA test followed by the Bonferroni post hoc test. No significant differences were found.

NfL protein expression was immunohistochemically assessed in the same areas as described for Ng. The observed immunolabeling pattern was characterized by a consistent staining of the neuropil and grey matter and intracytoplasmic staining in some neurons, especially located in dendrites. This immunostaining pattern was observed in the three analyzed groups, although higher immunolabeling intensities were found in the control group, and lower intensities were observed in the clinical group in all of the studied brain areas (Figure 2A).

The immunolabeling intensity was measured using Image J software (Rasband, W.S., U. S. National Institutes of Health, Bethesda, MD, USA), and the assessment of the differences between groups was performed using the one-way ANOVA test in each brain area (Figure 2B). In all of the assessed brain areas, the immunostaining intensity for NfL was higher in the control group, followed by preclinical animals and clinical animals, which exhibited the lowest intensities. After performing statistical analyses, significant differences were observed between negative and clinical animals in the obex, mesencephalon, thalamus, parietal cortex, basal ganglia, and frontal cortex (*p* < 0.01). Negative and preclinical animals showed significant differences in the thalamus and obex (*p* < 0.05), and clinical and preclinical sheep in the mesencephalon and parietal cortex (*p* < 0.05).

The specificity of the anti-NfL (M076229-2, Dako Agilent, Glostrup, Denmark) antibody against NfL in sheep nervous tissue was tested by Western blot in the frontal cortex of preclinical, clinical, and control sheep, revealing a single band at ~68 kDa, corresponding to NfL (Figure 2C).

Quantitative PCR was performed to assess the expression of *NEFL*, the gene that encodes the NfL protein, in the four brain areas previously used to study *NRGN* expression (obex, Hp, Th, and Fc) in the three study groups. Figure 2D shows the mean ∆Ct values of *NEFL*. Although no significant differences were found between groups, in the obex and thalamus. we can observe a tendency toward the downregulation of *NEFL* as the disease progressed. Similar levels of expression were found in the frontal cortexes of clinical, preclinical, and control sheep.

### 2.3. Scrapie-Related Neuropathological Features in Sheep and Correlation with Ng and NfL Protein Expression

**Figure 3:**
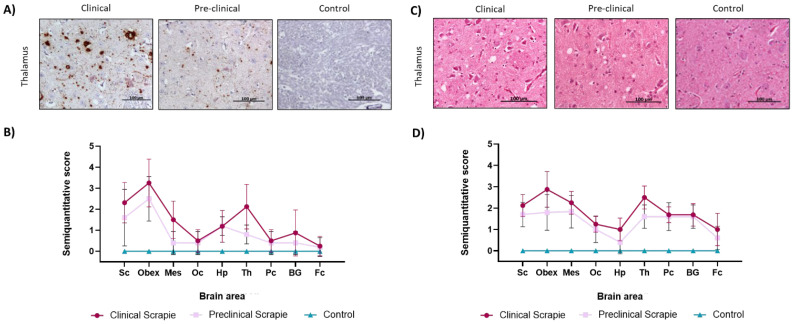
PrP^Sc^ deposition and spongiform changes in the brains of clinical (*n* = 8), preclinical (*n* = 5), and control (*n* = 8) sheep. (**A**) Representative images of PrP^Sc^ deposits detected by immunohistochemistry in the obex and thalamus of the three groups of sheep. (**B**) Graphics showing comparisons of the semiquantitative evaluation (on a scale of 0—lack of staining—to 5—staining present at maximum intensity) of these deposits and representing the mean ± SEM in the following areas: spinal cord (Sc), mesencephalon (Mes), occipital cortex (Oc), thalamus (Th), hippocampus (Hp), thalamus (Th), parietal cortex (Pc), basal ganglia (BG), and frontal cortex (Fc). (**C**) Representative images of hematoxylin and eosin staining in the obex and thalamus of clinical, preclinical, and control sheep. (**D**) Graphic results of the semiquantitative evaluation (on a scale of 0—lack of spongiosis—to 5—very intense spongiosis) of these lesions in the three sheep groups, representing the mean ± SEM, in the previously mentioned areas: spinal cord (Sc), mesencephalon (Mes), thalamus (Th), hippocampus (Hp), thalamus (Th), parietal cortex (Pc), basal ganglia (BG), and frontal cortex (Fc).

PrP^Sc^ deposition in sheep brains was evaluated by immunohistochemistry and semiquantitatively scored (from 0, meaning no PrP^Sc^ staining, to 5, meaning intense PrP^Sc^ immunolabeling) in the previously mentioned brain areas. All clinical and preclinical animals showed PrPSc deposition and spongiosis; therefore, all were included in the study of these features. As expected, negative controls showed no PrP^Sc^ deposition in the brain. The PrP^Sc^ deposition pattern was similar between the clinical and preclinical sheep, although the preclinical group showed lower scores. Peak scores were detected in the obex and thalamus, agreeing with previous descriptions of the PrP^Sc^ spreading pattern in sheep scrapie [31]. The results are shown in Figure 3A,B.

Spongiosis, which is the main histopathological lesion in prion diseases, was also semi-quantitatively evaluated in the same areas of the three groups of sheep on a scale from 0, meaning an absence of vacuoles, to 5, meaning intense spongiosis. No spongiosis was reported in the control group, while the clinical and preclinical groups presented vacuoles in the neuropil and neurons of all brain areas (Figure 3C). As observed in the PrP^Sc^ deposition, clinical and preclinical animals displayed similar spongiosis patterns, which were slightly lower in preclinical animals, and both groups showed higher scores in the obex and thalamus (Figure 3D).

Spearman’s ρ correlation was calculated in four brain areas (obex, hippocampus, thalamus, and frontal cortex) between the immunohistochemical scores for Ng and NfL and the scores of the neuropathological prion-associated features (PrP^Sc^ deposition and spongiosis) in all sheep at once to determine whether there was a correlation between prion-related lesions and the expression of these proteins. Correlation values and statistical significances can be found in Table 1.

As expected, PrP^Sc^ deposition and spongiosis displayed a positive and significant correlation in all of the studied brain areas. Regarding Ng expression, a negative correlation was found with both PrP^Sc^ and spongiosis. Spongiosis and Ng were negatively correlated in all of the studied areas, with this correlation being significant in three of them: obex, thalamus, and frontal cortex. Ng expression and PrP^Sc^ deposition also showed a negative significant correlation in the obex and thalamus.

NfL also displayed negative correlations with the scrapie-related neuropathological features, and this correlation was significant in the obex and thalamus. The association between the Ng and NfL protein expressions was also assessed, and a positive correlation was found, which was significant in the thalamus and obex.

### 2.4. Ng and NfL Concentrations in Sheep CSF

The Ng and NfL concentrations in ovine CSF were studied using two in-house ELISA tests as previously described [32,33]. The NfL ELISA test (based on the NfL21 and NfL23 antibodies) was able to detect sheep NfL. However, Ng determinations could not be performed, since the ELISA based on the Ng2 and Ng36 antibodies was not able to detect ovine Ng, probably due to the differences between the Ng2 antibody and the amino acid sequence of sheep Ng.

**Figure 4:**
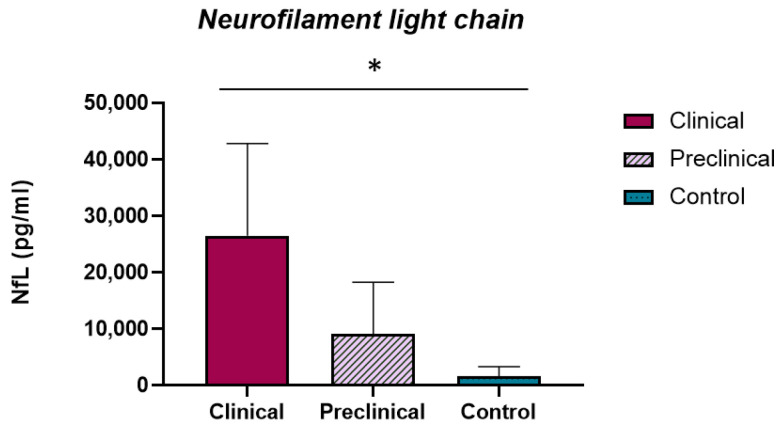
Analysis of CSF NfL levels in scrapie-infected and non-infected sheep. Clinical (*n* = 8), preclinical (*n* = 5), and control (*n* = 8) groups are shown. Representations of the NfL concentrations (pg/mL) found in the CSF of the three study groups are shown. Differences between groups were studied using the one-way ANOVA test followed by the Bonferroni post hoc test. NfL concentration in CSF was significantly different between the clinical and control groups (* *p* < 0.05). Data are expressed as the mean ± SEM.

Regarding the NfL concentrations in sheep CSF, the highest levels were detected in the clinical group of sheep (30,250 ± 6240 pg/mL), followed by the preclinical animals (9118 ± 3145 pg/mL), and finally the control group (775 ± 131 pg/mL) (Figure 4). The NfL concentrations were significantly different in the clinical versus control groups (*p* < 0.05).

## 3. Discussion

Prion diseases are fatal neurodegenerative disorders in which the main pathogenic event is the conversion of PrP^C^ into PrP^Sc^, leading to the accumulation of PrP^Sc^ in the central nervous system and causing neuronal dysfunction and cell death, which results in spongiform degeneration. The infection of the nervous tissue by prions is characterized by extensive spongiform degeneration with massive neuronal loss, synaptic alterations, neuroinflammation, and the accumulation of protein aggregates [34]. This process of neuronal loss begins with the presence of PrP^Sc^, causing changes in neurons associated with stress and a glial response. The damage progresses and alters synaptic transmission, which leads to neuronal death [29].

One of the ways in which prion-induced neurodegeneration might affect the synapse is through the loss of function of PrP^C^. Hitherto, the physiological role of PrP^C^ is still unclear, since no anatomical or developmental impairment has been observed in many previous studies using animal models with PrP^C^ depletion [35,36]. Nevertheless, physiological alterations have been reported in some PrP^C^-ablated models, including abnormalities in synaptic transmission, specifically in cognition [37]. Moreover, PrP^C^ has been localized at both pre- and post-synaptic membranes, and on synaptic vesicles [38,39], and it has also been found to interact with different proteins that take part in synaptic release [40]. These findings further support its role in neuronal synaptic transmission and synaptic plasticity [41].

The other way through which prion-induced pathogenesis could affect the synapse is the endoplasmic reticulum (ER) pathway. Different studies have demonstrated that the accumulation of PrP^Sc^ induces ER stress [42,43], activating pro-apoptotic pathways [44]. ER stress alters Ca^2+^ homeostasis, which regulates calcineurin (CaN), a type 2 phosphatase involved in synaptic function, memory, and cell death [45], and it has been proven that PrP^Sc^ accumulation causes synaptic dysfunction via CaN dysregulation [29].

Ng and NfL are synaptic and axonal markers that could reflect these alterations in prion diseases. After CNS axonal and synaptic damage, Ng and NfL are released to the interstitial fluid [9,24], which directly communicates with the CSF. Therefore, in this study, we assessed the expression of these two markers at the protein and gene levels in the brains of sheep throughout the pathogenesis of natural scrapie.

### 3.1. Ng Expression in Brain Tissue Was Affected by Prion-Related Pathogenesis

Reductions in Ng expression in preclinical scrapie-affected sheep as compared with negative controls were significant in several brain areas, such as the thalamus and obex. This could be explained by the fact that the thalamus and obex are among the first regions where PrP^Sc^ accumulates in the CNS [46]. The results obtained in the clinical and control groups of this study agree with those of previous studies in which Ng was evaluated as a biomarker of neurodegeneration in humans affected by CJD. The Ng levels were found to be significantly decreased in the brains of CJD patients compared with healthy controls, as assessed by immunohistochemistry [18]. As previously mentioned, scrapie, where preclinical naturally infected animals can be detected, is a model that allows comparative pathology to assess biopathological markers in the early stages of prion disease. In this study, clinical and preclinical animals showed very similar levels of Ng expression in all of the brain areas evaluated, thus agreeing with the hypothesis that synaptic damage is an early event in natural scrapie previously proposed for other natural and experimental prion diseases [18,30]. We also observed that the expression levels of Ng were lower in the preclinical group compared with the control group in all brain areas but the hippocampus. It is true that, since the preclinical animals included in this study were naturally infected, it is not possible to know exactly in which stage of infection they were in at the moment of culling. However, all preclinical animals came from the same herd, were within the same age, and showed similar neuropathological characteristics; hence, we believe they are a representative preclinical group for the study. Nevertheless, further studies using prion-inoculated transgenic mouse models expressing ovine PrP would be useful to confirm the results found in this study for both Ng and NfL.

To complement the protein expression studies, we assessed *NRGN* gene expression in four brain areas. In the frontal cortex, thalamus, and obex, the expression of the *NRGN* gene was decreased in clinical and preclinical animals compared with controls, and this downregulation was stronger in the clinical group. In the hippocampus, however, the controls showed a downregulation of the Ng-encoding gene compared with the other groups, while the preclinical animals exhibited an upregulation. All of these results agree with those found in the assessment of protein expression by immunohistochemistry. Susceptibility to prion infection varies between different brain areas [47,48]; therefore, this could explain the differences found in the hippocampus, since neuropathological changes in this area were less intense than those observed in the rest of the brain. Moreover, the results of recent studies in murine prion models suggest that the hippocampus is more resistant to prion infection than other brain regions, such as the thalamus and brainstem [49].

### 3.2. NfL Expression in Brain Tissue Was Reduced throughout the Course of the Disease

The results of NfL immunohistochemical evaluation were very similar to those found for Ng. The immunostaining was higher in the control group in all brain areas, followed by preclinical animals and then clinical animals. As described for Ng, the differences in NfL protein expression between the control and preclinical groups were higher and significant in brain areas showing more severe neurodegeneration (i.e., the thalamus and obex). These results, especially the significant differences found between the preclinical and the control group, agree with the statement that axonal damage is an early event in prion diseases [50].

Regarding the *NEFL* gene expression, in the areas of the frontal cortex, thalamus, and obex, this gene showed a downregulation trend in clinical and preclinical animals compared with controls, this being more pronounced in clinical animals. However, in the hippocampus, the controls showed the lowest *NEFL* expression levels, while the preclinical animals exhibited the highest expression levels. These results found in the hippocampus disagree with those found for NfL protein expression in the same brain area. *NEFL* mRNA has been found to be altered in other neurodegenerative diseases, such as ALS [51,52], and this finding in the hippocampus supports a different response in this area to prion-associated pathogenesis.

### 3.3. Ng and NfL Levels in Brain Tissue Are Negatively Correlated with Prion-Associated Lesions

The results of the correlation analyses between scrapie-related neuropathological features in sheep and Ng and NfL proteins showed, as expected, that there is a relationship between the present quantity of the studied proteins in the brains and prion-associated neurodegeneration. Moreover, the thalamus and the obex, which are, as previously mentioned, two of the brain areas earlier and more severely affected by prions [46], showed a strong significant negative correlation between the histopathological markers and Ng and NfL expression, indicating that the neuropathological phenomena occurring in prion diseases directly influence the quantity of these proteins in brain tissue. This negative correlation between Ng and NfL and prion pathology detected by immunohistochemistry could be, in part, due to the neuronal loss occurring in the infected animals. However, the qPCR results also showed decreases in Ng and NfL expression in the preclinical and clinical sheep. In addition, we also detected an increase in NfL in the CSF; therefore, we do not believe that the reduction of staining found in immunohistochemistry was only due to the neuronal loss that develops in prion diseases.

### 3.4. NfL Levels Are Increased in the CSF of Scrapie-Affected Sheep

Regarding the CSF tests, even though no statistical differences were observed, the CSF NfL mean values were higher in preclinical sheep compared with controls, in agreement with the previous results found for 14-3-3 and tau [8]. This again supports that the underlying neurodegenerative mechanisms of prion diseases can also occur in the asymptomatic stages of the disease and, although NfL is an unspecific marker of axonal damage, it could be useful in the identification of preclinical prion cases combined with other proteins, such as tau and 14-3-3. Unfortunately, Ng measurements in the CSF could not be performed. However, our results for Ng expression in the brain tissue suggest Ng as a useful tool for the identification of preclinical cases of prion diseases. Ng has been reported to be increased in the CSF of human clinical cases of AD and sCJD, this being an stronger increase in sCJD compared with AD [18]. Therefore, although other neurodegenerative diseases could also affect the levels of Ng both in the brain tissue and the CSF, it could be used in combination with other biomarkers, such as 14-3-3 or tau, also found to be higher in the CSF in preclinical cases of scrapie [8]. This opens a valuable path in research for the development of tests capable of measuring Ng in CSF to assess its value as a preclinical biomarker.

### 3.5. Conclusions

This study, as with others evaluating preclinical biomarkers of prion disease [8], focused on scrapie. However, these results could be extrapolated to human prion diseases. The identification of biopathological markers allowing the detection of prion disease cases in the asymptomatic stages, in which the neuronal damage is not yet massive, is needed to increase the efficiency of possible treatments whenever these are available. In this study, we corroborated that the concentration of two markers of synaptic and axonal damage, Ng and NfL, in brain tissue is affected by prion accumulation, and that this condition is noticeable, even in preclinical stages of prion-associated neurodegeneration. Therefore, although further research is needed, the obtained results strongly support the usefulness of Ng and NfL as preclinical biomarkers for prion diseases.

## 4. Materials and Methods

### 4.1. Animals and Sample Collection

A total of 21 female *Rasa Aragonesa* sheep, aged between 4 and 6 years, were included in this study. These animals belonged to three different groups in terms of their classical scrapie condition: clinical, preclinical, and uninfected. All animals displayed an ARQ/ARQ genotype regarding the *PRNP* gene. Control animals (*n* = 8) were selected from a herd in which no scrapie cases had been reported. Scrapie-infected animals, both clinical (*n* = 8) and preclinical (*n* = 5), were obtained from two different scrapie-affected herds. Characteristics from each animal are detailed in the supplementary materials (Table S1).

Sheep in the preclinical stage were identified by immunohistochemical analyses of rectal mucosa biopsies [4] and sacrificed by pentobarbital overdose before clinical signs were detectable. Animals in the clinical stage were identified by the observation of scrapie-associated clinical signs, which included pruritus and scratching of the tail root, lumbar area, and limbs, neurological signs, such as ataxia and head tremors, teeth grinding, wool loss, and cachexia. Clinical animals were confirmed to be scrapie-positive by the detection of PrP^Sc^ in the brain by immunohistochemical analyses.

Once the animals were sacrificed, two replicate samples from nine different areas of the CNS (frontal cortex, basal ganglia, parietal cortex, thalamus, occipital cortex, hippocampus, mesencephalon, obex, and spinal cord) were collected in formaldehyde (10%) and embedded in paraffin for further use in histopathological and immunohistochemical analyses or preserved by freezing for further biomolecular analyses. In addition, samples from four areas (frontal cortex, thalamus, hippocampus, and obex) were collected in RNAlater^TM^ solution (Thermo Fisher Scientific, Waltham, MA, USA) for use in gene expression analyses, and 1 mL to 1.5 mL of the CSF from each animal was collected and conserved by freezing at −80 °C for further analyses.

### 4.2. Histological and Immunohistochemical Analyses

To perform immunohistochemical and histopathological studies, paraffin-embedded sections from the nine previously mentioned areas (4 μm thick) were used. Neuropathological changes and spongiform lesions were studied by hematoxylin–eosin staining. PrPSc was detected using the monoclonal primary antibody L42 (1:500, R-Biopharm, Darmstadt, Germany) after formic acid treatment and proteinase K digestion, as previously described [53].

Immunohistochemistry for the detection of Ng and NfL was performed after antigen retrieval with citrate buffer (pH 6.0) for 20 min at 96 °C, and endogenous peroxidase activity blocking using a precast solution (Dako Agilent, Glostrup, Denmark). Sections were incubated with the primary antibodies Ng36 (1:400; in-house generated, Department of Psychiatry and Neurochemistry, Mölndal, Sweden [33]) or anti-NfL (1:500, Dako Agilent, Glostrup, Denmark, M076229-2) overnight at 4 °C. Then, the samples were incubated with an anti-mouse enzyme-conjugated Envision polymer (Dako Agilent, Glostrup, Denmark) for 30 min at room temperature (RT). DAB (diaminobenzidine, Dako Agilent, Glostrup, Denmark) was used as the chromogen. To reduce possible staining intensity differences due to the immunohistochemistry performance, all animals were stained in a single batch for each marker and area. Negative controls were performed both by using an IgG1 isotype control antibody (mouse IgG1, kappa monoclonal (MOPC-21)—isotype control, ab18443, Abcam, Cambridge, UK) and by the omission of the primary antibody.

Brain sections were examined using a Zeiss Axioskop 40 optical microscope (Zeiss, Jena, Germany). All samples were blindly analyzed. Spongiosis and PrP^Sc^ deposition were semiquantitatively evaluated twice per area by scoring on a scale of 0 (lack of spongiosis/PrP^Sc^ deposit) to 5 (very intense spongiosis/PrP^Sc^ deposit) following the standard method to assess these features [54]. Ng and NfL immunostainings were assessed using the Image J software. Two photographs of each brain area per sheep were evaluated. The Image J color deconvolution method, measuring both the number of stained cells and the immunostaining intensity, was used as previously described [55].

### 4.3. Cerebrospinal Fluid Analyses

CSF NfL concentration was measured using an in-house enzyme-linked immunosorbent assay (ELISA) based on the NfL21 and NfL23 monoclonal antibodies, as described in detail elsewhere [32]. An in-house ELISA based on the Ng2 and Ng36 monoclonal antibodies, as previously described in detail [33], was used for CSF Ng measurement. The measurements were performed in one round of experiments by a board-certified laboratory technician who was blinded to diagnostic information. Intra-assay coefficients of variation were below 10%.

### 4.4. Western Blot Analyses

Twenty-five micrograms of total protein from the frontal cortex of four sheep per group (clinical, preclinical, and controls) were subjected to electrophoresis in 10% SDS/PAGE gels and transferred to PVDF membranes (Bio-Rad, Hercules, CA, USA). After blocking at room temperature for 1 h with 2% bovine serum albumin (Thermo Fisher Scientific, Waltham, MA, USA), the membranes were incubated overnight at 4 °C with the primary antibodies Ng36 (1:2000; in-house generated, Department of Psychiatry and Neurochemistry, Mölndal, Sweden) and anti-NfL (1:1000, Dako Agilent, Glostrup, Denmark, M076229-2) against Ng and NfL, respectively, diluted in PBST (1X phosphate-buffered saline, 0.1% Tween). After that, the membranes were incubated for 1 h at RT with an HRP-conjugated secondary antibody diluted 1:5000 in PBST (goat anti-mouse IgG-HRP, Santa Cruz Biotechnology, Dallas, TX, USA). Membranes were washed and then the signal was developed using the ECL Plus Western Blotting system (GE Healthcare, UK) and visualized with the VersaDoc imaging system (Bio-Rad, Hercules, CA, USA).

### 4.5. Gene Expression Analyses

The expression profile of the genes neurogranin (*NRGN*) and neurofilament light chain (*NEFL*) was assessed in four brain areas (frontal cortex, thalamus, hippocampus, and obex). The total RNA was obtained from 100 mg of tissue preserved in RNAlater™ Solution (Thermo Fisher Scientific, Waltham, MA, USA) using an RNeasy Lipid Tissue Mini kit (QIAGEN^®^, Venlo, The Netherlands) following the manufacturer’s recommended protocol.

Complementary DNA (cDNA) was obtained from 1 µg of total RNA using qScript™ cDNA Supermix (Quanta Biosciences™, Beverly, MA, USA), and the resulting cDNA was diluted 1:5 in pure water for further analyses.

Primers for *NRGN* and *NEFL* were designed by using the Primer3Plus tool [56]. To make them cDNA-specific, the primers were designed to expand exon–exon junctions.

The quantitative real-time polymerase chain reaction (qPCR) was performed using SYBR^®^ Green master mix (Applied Biosystems, Waltham, MA, USA) and carried out in a StepOne Real-Time PCR System (Thermo Fisher Scientific, Waltham, MA, USA) using universal conditions. All reactions were run in triplicate in a total volume of 10 μL using 2 μL of diluted cDNA. The expression of the housekeeping genes GAPDH and SDHA was measured using previously described primer sequences and conditions [57] and used to normalize the results. Relative gene expression quantification was then performed using the 2^−∆∆Ct^ method. The sequences, accession numbers, and concentrations of the used primers can be found in Table 2.

### 4.6. Data Analysis and Statistics

SPSS (SPSS Statistics for Windows, Version 17.0, Chicago, IL, USA) software was used for the statistical analyses. Graphs were generated with GraphPad Prism 8.0 (San Diego, CA, USA). All quantitative data were tested for normality with the Shapiro–Wilk W test. Between-group differences in histological and immunohistochemical scores, as well as in the CSF protein levels, were determined using the one-way analysis of variance (ANOVA) test followed by the Bonferroni post hoc test. Statistical analyses of the qPCR data were conducted by comparing the mean ΔCt values for each gene. For the statistical comparison, the one-way ANOVA test followed by the Bonferroni post hoc test was also used. Differences between groups were considered to be significant at *p* < 0.05. Spearman’s ρ correlation was used to assess the correlation between prion-related lesions and the expression of the studied proteins.

## Figures and Tables

**Table 1:** Spearman’s correlation values between scores of Ng and NfL expression and prion-associated histopathological lesions in different brain areas.

		Ng	NfL	PrPSc	Spongiosis
**Obex**	**Ng**	-	0.4918 *	−0.6006 **	−0.7227 ***
	**NfL**	0.4918 *	-	−0.7755 ****	−0.7497 ***
	**PrP^Sc^**	−0.6006 **	−0.7755 ****	-	0.8633 ****
	**Spongiosis**	−0.7227 ***	−0.7497 ***	0.8633 ****	-
**Hp**	**Ng**	-	0.2959 ^n.s.^	−0.2786 ^n.s.^	−0.2238 ^n.s.^
	**NfL**	0.2959 ^n.s.^	-	−0.2145 ^n.s.^	−0.2313 ^n.s.^
	**PrP^Sc^**	−0.2786 ^n.s.^	−0.2145 ^n.s.^	-	0.7921 ****
	**Spongiosis**	−0.2238 ^n.s.^	−0.2313 ^n.s.^	0.7921 ****	-
**Th**	**Ng**	-	0.6994 ***	−0.8124 ****	−0.8038 ****
	**NfL**	0.6994 ***	-	−0.8130 ****	−0.6530 **
	**PrP^Sc^**	−0.8124 ****	−0.8130 ****	-	0.8112 ****
	**Spongiosis**	−0.8038 ****	−0.6530 **	0.8112 ****	-
**Fc**	**Ng**	-	0.2240 ^n.s.^	−0.2587 ^n.s.^	−0.5540 **
	**NfL**	0.2240 ^n.s.^	-	−0.1579 ^n.s.^	−0.3233 ^n.s.^
	**PrP^Sc^**	−0.2587 ^n.s.^	−0.1579 ^n.s.^	-	0.4971 *
	**Spongiosis**	−0.5540 **	−0.3233 ^n.s.^	0.4971 *	-

Spearman’s correlation * *p* < 0.05, ** *p* < 0.01, *** *p* < 0.001, **** *p* < 0.0001, and n.s.: no statistically significant value. Ng: Neurogranin; NfL: Neurofilament light chain; Hp: Hippocampus; Th: Thalamus; Fc: Frontal cortex.

**Table 2:** Primers used in the gene expression analyses.

Gene	Forward (F) and Reverse (R) Primer Sequence (5′-3′)	Concentration (nM)	Accession Number
*NRGN*	F AACCTCCATCCCAGCCAAG	400	XM_027959688.2
	R CCCGGAAACTCGCCTGTA	400	
*NEFL*	F CATTAGCGCTATGCAGGACA	400	XM_015093090.3
	R GCTGCAATCTCAATGTCCAA	400	
*GAPDH*	F TCCATGACCACTTTGGCATCGT	900	AF_035421
	R GTCTTCTGGGTGGCAGTGA	900	
*SDHA*	F CATCCACTACATGACGGAGCA	300	AY_970969
	R ATCTTGCCATCTTCAGTTCTGCTA	300	

## Data Availability

The data presented in this study are available within the article’s text and figures.

## Supplementary Materials

The following supporting information can be downloaded at: https://www.mdpi.com/article/10.3390/ijms23137182/s1.
