# Supplementary material for: Neurogranin and Neurofilament Light Chain as Preclinical Biomarkers in Scrapie
Source: Int J Mol Sci. 2022 Jun 28;23(13):7182. doi: 10.3390/ijms23137182 (PMC9266981; doi:10.3390/ijms23137182)
Supplement: Supplementary file 1 [file ijms-23-07182-s001.zip › ijms-1774353-supplementary.pdf]

# Supplementary materials

**Table S1.** Demographic details of the animals included in the study.

| Specie | Group | ID | *PRNP* polimorfism | Herd | Age at culling |
|---|---|---|---|---|---|
| **Sheep** (*Ovis aries*) | Negative | 1 | ARQ/ARQ | A | 4 |
| | | 2 | ARQ/ARQ | A | 4 |
| | | 3 | ARQ/ARQ | A | 4 |
| | | 4 | ARQ/ARQ | A | 5 |
| | | 5 | ARQ/ARQ | A | 5 |
| | | 6 | ARQ/ARQ | A | 5 |
| | | 7 | ARQ/ARQ | A | 6 |
| | | 8 | ARQ/ARQ | A | 6 |
| | Preclinical | 9 | ARQ/ARQ | B | 4 |
| | | 10 | ARQ/ARQ | B | 4 |
| | | 11 | ARQ/ARQ | B | 4 |
| | | 12 | ARQ/ARQ | B | 4 |
| | | 13 | ARQ/ARQ | B | 5 |
| | Clinical | 14 | ARQ/ARQ | C | 4 |
| | | 15 | ARQ/ARQ | C | 4 |
| | | 16 | ARQ/ARQ | C | 5 |
| | | 17 | ARQ/ARQ | C | 5 |
| | | 18 | ARQ/ARQ | C | 5 |
| | | 19 | ARQ/ARQ | C | 6 |
| | | 20 | ARQ/ARQ | C | 6 |
| | | 21 | ARQ/ARQ | C | 6 |

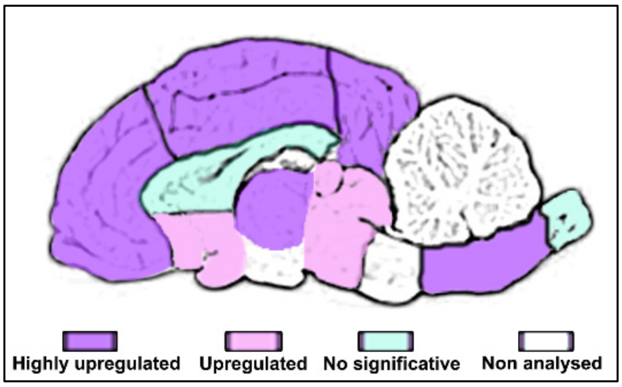


**Figure S1.** Schematic map of the brain showing the degree of Ng upregulation in control animals.
